# Post-Omicron SARS-CoV-2 antibody prevalence in Sierra Leone: A cross-sectional, nationally representative, follow-up serosurvey

**DOI:** 10.1371/journal.pgph.0004273

**Published:** 2025-04-16

**Authors:** Smit Chitre, Mohamed Bailor Barrie, Joseph Sam Kanu, Theophilus S. Conteh, Mohamed Bayoh, Matilda N. Kamara, Haja Fatmata Bangura, Jonathan S. Lascher, Raphael Frankfurter, Sarah A. Goldberg, David V. Glidden, J. Daniel Kelly, Sulaiman Lakoh, Eugene T. Richardson

**Affiliations:** 1 Department of Global Health and Social Medicine, Harvard Medical School, Boston, Massachusetts, United States of America; 2 Partners In Health, Kono, Sierra Leone; 3 Institute for Global Health Sciences, University of California, San Francisco, California, United States of America; 4 Ministry of Health and Sanitation, Government of Sierra Leone, Freetown, Sierra Leone; 5 College of Medicine and Allied Health Sciences, University of Sierra Leone, Freetown, Sierra Leone; 6 David Geffen School of Medicine, University of California, Los Angeles, Los Angeles, California, United States of America; 7 Department of Medicine, Brigham and Women’s Hospital, Boston, Massachusetts, United States of America; 8 Department of Epidemiology and Biostatistics, University of California, San Francisco, California, United States of America; Ashoka University, INDIA

## Abstract

Based on a serosurvey conducted in March 2021, Barrie and colleagues published the first nationally representative SARS-CoV-2 serosurvey in Africa, estimating a SARS-CoV-2 seroprevalence of 2.6% in Sierra Leone, 43 times higher than the reported number of cases at that time. Over the following two years, increasingly transmissible variants—specifically Delta and Omicron—proliferated across the globe, and their impact in Africa is poorly understood. Additional nationally representative seroprevalence data are therefore necessary to understand the pandemic’s progression on the continent and for evaluating containment measures and future preparedness. Our follow-up nationally representative survey was conducted in Sierra Leone from February to March 2023. We returned to the 120 Enumeration Areas throughout the country collecting blood samples from one or more individuals per household as well as information on sociodemographic characteristics, history of COVID-19 infection and immunization, and attitudes towards vaccination. The weighted overall seroprevalence (vaccinated and/or SARS-CoV-2 infection) for individuals >19 years of age was 33% (95% CI 29–37). Using the data and distributions from our previous serosurvey, the weighted predicted seroprevalence (any prior SARS-CoV-2 infection) for the general population was 28% (95% CI 15–41). The weighted predicted seroprevalence was ~11 times higher than the pre-Delta/Omicron prevalence. It was also over 300 times higher than the reported number of cases. Despite this, overall seroprevalence was low compared with countries in Europe and the Americas (pointing towards lower transmission in Sierra Leone). In addition, our results suggest the following regarding prevention campaigns claiming to have vaccinated 70% of adults in Sierra Leone as of December 2022: 1) they resulted in limited seroconversion; 2) there was significant waning of immunity; and/or 3) many less individuals were vaccinated than reported. Regardless of the cause, the utility of COVID-19 Vaccine Delivery Partnership (CoVDP) efforts three years into the pandemic is called into question.

## Introduction

**Fig 1:**
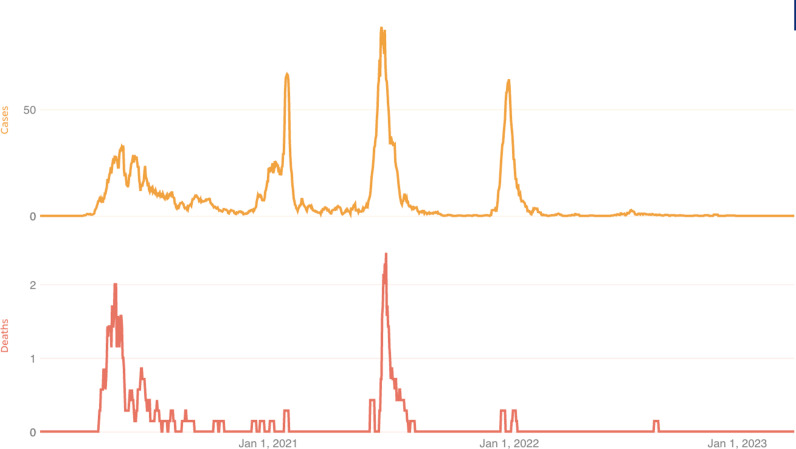
Daily *reported* COVID-19 cases and deaths in Sierra Leone from 23 January 2020 to 5 March 2023 (Source: Johns Hopkins University & Medicine Coronavirus Resource Center. Accessed 7 March 2023).

As of 5 March 2023, the National Disease Surveillance Program in Sierra Leone reported 7760 cases (92 per 100,000 population) and 126 deaths due to COVID-19 (Fig 1). Despite multiple waves of increasingly transmissible variants worldwide [1], the case rate in Sierra Leone appears to have remained relatively low. The ostensibly limited impact of the pandemic on the African continent—at least according to *reported* infections and deaths—has been be attributed to the decisive implementation of stay-at-home mandates, effective sensitization via community health workers, and an overarching COVID-19 mitigation strategy developed on the continental level by the Africa Centres for Disease Control and Prevention (Africa CDC) [2–5].

Due to under-resourced active surveillance and a dearth of nationally representative serosurveys, however, our understanding of SARS-CoV-2 incidence and prevalence across the African continent remains limited. We searched PubMed using the terms (“SARS-CoV-2” [Title/Abstract]) AND (“seroprevalence” [Title/Abstract]) AND (“Africa”) on December 21, 2023, with no date restrictions, and received 140 results. Only three nationally representative SARS-CoV-2 seroprevalence studies were identified (South Africa, the Republic of the Congo, and Sierra Leone). Three systematic reviews conducted prior to the spread of the Omicron variant reported a range of continental seroprevalence estimates: In a meta-analysis spanning 35 seroprevalence studies conducted in Africa, Hajissa and colleagues reported a SARS-CoV-2-antibody seroprevalence of 16% [6]; Chisale et al. determined a pooled seroprevalence of 22% (with very high heterogeneity) for the continent as of mid-2021; and Lewis et al. estimated this figure at 65.1% from July to December 2021 [7,8].

Within Sierra Leone, we published the first nationally representative serosurvey on the continent and calculated a weighted seroprevalence of 2.6% in March 2021–43 times higher than the number of reported cases at that time [9]. We observed that event-based reporting methods in Sierra Leone tended to rely on under-resourced strategies for contact tracing and case detection, leading to under-reporting of the true burden of viral disease [10]. In the wake of increasingly more contagious SARS-CoV-2 variants [11,12]—and since no nationally representative, pre- and post-Omicron SARS-CoV-2 serosurveys had been conducted on the African continent—we investigated the change in SARS-CoV-2 seroprevalence after the Delta and Omicron waves in Sierra Leone, as this would have implications for evaluating public health responses and for future policy planning, both on national and continental levels [13–15].

## Methods

### Design

In this population-based, cross-sectional, follow-up seroprevalence survey, we tested individuals aged 5 years and older for the presence of SARS-CoV-2 IgM and IgG. Participants (Table 1) were tested regardless of previous or current infection with COVID-19 but were required to have resided in Sierra Leone during the period of SARS-CoV-2 transmission in the country (i.e., since 31 March 2020, when the first case was reported).

**Table 1:** Participant characteristics.

	Participants (n=1,268)
Age, years	
< 10	233 (18.4%)
10–19	288 (22.7%)
20–39	333 (26.3%)
40–59	235 (18.5%)
> 60	179 (14.1%)
Gender	
Female	599 (47.2%)
Male	669 (52.8%)
Number of People Per Household (n = 1,263)^§^	7 (5-10)* (1-30)^†^
Seropositive	381 (30.0%)
Positive IgM	6 (0.5%)
Positive IgG	377 (29.7%)

*median (interquartile range)

† (absolute range)

§ Data are n (%) unless otherwise stated

This serosurvey follows up on the nationally representative survey we conducted in March 2021. In the previous survey, we considered a seroprevalence estimate of 5% with 95% confidence intervals, a ± 2% overall confidence limit, and a design effect of 3, which produced a minimum sample size of 1200 households.

### Sampling

Our sampling frame was the 2015 census from Statistics Sierra Leone [16]. For the first survey, we derived our sampling strategy from the Africa CDC’s generic protocol for population-based, age and gender stratified serosurveys of SARS-CoV-2 [17]. Our randomized, multistage sampling process involved first randomly selecting 120 Enumeration Areas (EAs), which are geographical units each containing 80–120 households. EAs were randomly selected on a national level, not according to district weight. In each EA, we assigned numbers to all households distinguishable on a satellite map by labeling them in numerical order from west to east, and north to south, akin to other peer-reviewed studies [18–20]. Using a random number generator, we then selected ten households to survey per EA for a total of 1200 households.

For this follow-up survey, we used the same EAs, but we sampled a new set of households by running the random number generator once again. We tested and surveyed one or more members of each selected household. In order to decrease the association between individuals included in the overall sample, we kept the number of individuals from a single cluster relatively small; however, since many adults had reportedly been vaccinated in the year prior to the follow-up serosurvey, the selection of participants was modified to oversample household members between 5–10 years old (i.e., those that had not been vaccinated). Participants were recruited from 13 February 2023 to 3 March 2023.

### Test validation and quantitative data collection

Each fingerstick sample was screened for both IgM and IgG SARS-CoV-2-specific antibodies (spike receptor-binding domain) with the Hangzhou Biotest Biotech RightSign COVID-19 Rapid Test Cassette, which was the assay recommended and procured by the Africa CDC for our first serosurvey. This particular antibody test was shown by U.S. Food and Drug Administration (FDA) to have combined IgM/IgG specificities of 100% and IgM/IgG sensitivities of 100% [21].

We had further validated the test in the previous survey with a control panel of 58 serum samples (from 18 individuals tested by nasopharyngeal swab—10 who tested negative and 8 positive, with 5 serial dilutions of each positive) and also found combined IgM/IgG sensitivities and specificities of 100% [9].

Fingerstick tests were performed simultaneously with participant interviews and in accordance with the manufacturer’s instruction. Each participant was counseled before testing and informed of their results when the cassette revealed them.

### Qualitative data collection

Participant interviews entailed asking participants their age, sex, household size, and occupation. In addition, we asked participants to share their COVID-19 vaccination status, their reasons for choosing to take or not take the vaccine, and their general thoughts on vaccination as a countermeasure to the spread of the coronavirus. In the case of participants who were minors, we asked their parents or guardians to respond to demographic and health-related questions, but we did not collect subjective responses on vaccine decision-making.

### Quantitative data analysis

Data were analyzed to estimate an unweighted seroprevalence of IgM/IgG antibodies against SARS-CoV-2 with a 95% CI (Table 2, Column 3). Individuals were considered seropositive if they were IgM+/IgG−, IgM−/IgG+, or IgM+/IgG+. To estimate the weighted seroprevalence, we stratified by district (counting Falaba as part of Koinadugu) and calculated sampling weights as a ratio of the national population to the district population (Table 2, Column 4). Since these estimates included individuals ≥ 10 years old, 70% of whom had reportedly been vaccinated as of December 2022 [22], we used the results from individuals < 10 years (i.e., unvaccinated individuals) to predict weighted seroprevalences for the other age cohorts in the follow-up survey: A logistic regression model was fit among those < 10 years to estimate the odds ratio of seroprevalence by district/age. The odds ratios were combined with the district/age specific March 2021 serosurvey results in those ≥ 10 years of age to infer the counterfactual seroprevalence in the second survey (Table 2, Column 5).

**Table 2:** Seroprevalence by age cohort. *Weighted using inverse probability weights.

	Participants tested, n	Seropositive participants, n	Unweighted Seroprevalence, n (95% CI)	Weighted Overall Seroprevalence (Vaccinated and/or SARS-CoV-2 Infection), % (95% CI)	Weighted Predicted Seroprevalence (Any Prior SARS-Cov-2 Infection), % (95% CI)*
Overall	1,268	381	30% (28-33)	30% (27-32)	28% (15-41)
Age					
<10	233	46	20% (15-25)	19% (14-25)	19% (14-24)
10-19	288	90	31% (26-37)	30% (25-36)	26% (11-42)
>19	747	245	33% (29-36)	33% (29-37)	32% (15-49)

### Qualitative data analysis

Survey and interview data were coded and analyzed separately by two of the co-authors. The analyses were then consolidated to identify common themes. Codes were initially developed through an iterative process, starting with an open coding approach to capture emergent concepts, followed by refinement into a coding framework. Discrepancies between coders were resolved through discussion and consensus to ensure accuracy and reliability in theme identification.

### Ethics statement

After discussing a consent script, formal (witnessed and documented) verbal consent was obtained from all participants aged 18 years and older. Minors aged 5–17 years gave verbal assent to participate, and a parent, guardian, or caregiver gave formal verbal consent to enroll. The study was approved by the Sierra Leone Ethics and Scientific Review Committee. The qualitative component of the study was approved by the Harvard Institutional Review Board (IRB21–1142); however, the serosurvey received a Not Research Determination (IRB20–1394) from the Harvard IRB as it was deemed Public Health Surveillance.

## Results

In February/March 2023, 13 months after the Omicron wave peak (Fig 1), we identified 1268 individuals aged 5 years or older from households in 120 EAs. Two hundred and thirty-three (18.4%) participants were aged 5–9 years; 288 (22.7%) 10–19 years; 333 (26.3%) 20–39 years; 235 (18.5%) 40–59 years; and 179 (14.1%) >60 years. Five-hundred and ninety-nine of the participants (47.2%) were female, and 1044 (82.3%) were residing in rural areas (Table 1). The household response rate ranged from 90% to 100% in all districts except for Western Area, where around 25% of targeted households declined participation.

The weighted seroprevalence for unvaccinated <10-year-olds was 19% (95% CI 14–25). The weighted overall seroprevalence (vaccinated and/or SARS-CoV-2 infection) for individuals >19 years of age was 33% (95% CI 29–37). Using the data and distributions from our previous serosurvey, the weighted predicted seroprevalence (any prior SARS-Cov-2 infection) for the general population was 28% (95% CI 15–41).

Results from the qualitative study accompanying this seroprevalence survey identified distrust of public health campaigns as a major theme: In nearly every village, roughly 40% of individuals 1) wavered regarding their vaccination status (e.g., when asked to produce a vaccination card, they admitted to not being vaccinated); 2) were not vaccinated but would be willing to receive a vaccine if only they knew where to get one (many of these individuals stated they were at work, in school, or visiting another town when vaccines were available in their region); or 3) were outright unwilling to be vaccinated. Reasons for refusing the vaccine ranged from the belief that inoculation was only meant for “traveling people” to the concern that the “*chuk* will kill we [the vaccine will kill us].” Even nurses and community health workers, particularly in Western Sierra Leone, declined the vaccine out of fear of its side effects. Some health workers in Freetown cited an incident in which a nurse at Cottage Hospital died shortly after being vaccinated to justify their skepticism.

## Discussion

Based on a serosurvey conducted from February to March 2023, we estimated that 28% (95% CI 15–41) of Sierra Leone’s population had been previously infected with SARS-CoV-2. This is ~11 times higher than the pre-Delta/Omicron prevalence but still significantly lower than most serosurveys conducted in other countries after the Delta and Omicron waves [23,24]. It suggests that SARS-CoV-2 transmission in Sierra Leone, and perhaps other parts of West Africa, was not nearly as intense as surmised by epidemiologists [25]. This could be due to less indoor social mixing or underlying T-cell immunity to related coronaviruses that protects against seroconversion [26].

As there were only 7,760 reported cases of COVID-19 in Sierra Leone as of 3 March 2023 (Fig 1) [27], the data also suggest the true number of infections was at least 300 times higher than that identified by the health system. While a total seroprevalence of 28% is significantly lower than city and regional estimates across the continent, our studies are the first nationally representative pre-and-post Omicron SARS-CoV-2 serosurveys of an African nation to be published, avoiding potential biases found in other approaches. In a survey undertaken from October to November 2020 in Cameroon, Nwosu and colleagues found a seroprevalence of 29.2% in a Yaoundé health district [28]. From November 2020 to June 2021 (i.e., pre-Omicron), Moyo and colleagues estimated a seroprevalence of 37.8% (95% CI 35.4–40.4) in South Africa [29]. In Togo, as of June 2021, Konu and colleagues estimated a seroprevalence of 65.5% in 12 of 36 health districts [30]. Wondeu and colleagues estimated that 73.6% of students at the University of Cameroon were positive for SARS-CoV-2 antibodies in a study that spanned from December 2020 to December 2021 [31]. Kolawole and colleagues’ study of twelve states in Nigeria during the summer of 2021 estimated SARS-CoV-2 antibody prevalence at 78.9% [32]. A non-peer-reviewed preprint from Udoakang and colleagues reported SARS-CoV-2 IgG seropositivity ranging from 24.1 - 77.0% in Burkina Faso, Ghana, and Nigeria between June 2021 and June 2022. In eastern Uganda, Briggs and colleagues estimated that 67.7% of a rural cohort study population had been infected with SARS-CoV-2 pre-Omicron, while 84.8% of unvaccinated, previously seronegative individuals were infected post-Omicron [33]. In addition, using the same assay as in our survey, Ndziessi and colleagues estimated that, as of February 2022, 48.2% (95% CI 47.2–49.2) of the general population in the Republic of Congo had antibodies to SARS-CoV-2 after the first Omicron wave, but they did not distinguish between vaccination and infection [34]. Lastly, Osman and colleagues documented a SARS CoV-2 seroincidence of 84% in Bo, Sierra Leone using more robust antibody assays than in our survey; however, their study was limited to a single urban area, and it is unclear the investigators adjusted appropriately for prior vaccination [35].

As we conjectured previously, significant under-reporting of cases may be the result of a higher proportion of minimally symptomatic disease in a younger demography coupled with under-resourced systematic surveillance in the setting of legacies of ‘distrust’ and foreign extractivism (resulting in the avoidance of testing) [9,36–43]. Indeed, Mwananyanda and colleagues demonstrated significant under-reporting of cases in Zambia through postmortem surveillance [44].

Overall, our findings question the utility (and/or accounting) of CoVDP campaigns that claim to have vaccinated 70% of adults in Sierra Leone as of December 2022 [22]. (Internal accounting at the Sierra Leone Ministry of Health and Sanitation documents more [2^nd^] booster doses than 1^st^ doses in some districts, for example.) Vaccination campaigns conducted in Sierra Leone used a combination of the AstraZeneca, Sinovac, Johnson & Johnson, Pfizer, Sinopharm vaccines [45], the first four of which result in >90% seroconversion after primary vaccination (Sinopharm results in ~55% seroconversion) [46–50]. We found that only 33% (95% CI 29–37) of individuals >19 years old were seropositive (i.e., through vaccination, SARS-CoV-2 infection, or both), and we predicted that 32% (95% CI 15–49) would be seropositive without vaccination, suggesting that the vaccination campaigns in Sierra Leone resulted in limited seroconversion (e.g., there were issues with the vaccines), there was significant waning of immunity, and/or many less individuals were vaccinated than reported.

Our interviews with a randomized sample of adults across the country indicate some contribution from the interpretation that many less individuals were vaccinated than reported, especially given that most individuals voiced strong distrust of COVID-19 interventions (both lockdowns and patient-focused actions). Regardless of the cause, limited humoral immunity in a region reported to have the highest demography-adjusted infection fatality rates in the world corroborates earlier concerns about vaccine apartheid [51,52]. Further studies examining campaigns in other low-income countries may therefore be in order.

Our study has three notable limitations. First, our assay did not discriminate between infection and vaccination, forcing us to modify our methods to make estimates based on prevalence in children under 10 years old. As such, the results we report assume that age-stratified differences related to COVID-19 exposure and infection of COVID-19 over time remained constant, which may not be true. Furthermore, the fact that we were obliged to predict seroprevalence this time around caused the additional imprecision observed in adults. Second, the effects of waning immunity on the performance of the assay we used are unclear, and we therefore may not have fully captured those individuals infected in multiple pandemic waves (Fig 1). Lastly, the exact sensitivity of the assay for detecting minimally symptomatic infection is not known. The last two factors could have caused us to underestimate the true seroprevalence.

## Conclusion

While molecular surveillance in wastewater has been promoted as a means of providing near-real-time, comprehensive data on the burden of COVID-19 in communities, Sierra Leone has only a single wastewater treatment plant that serves less than 1/8 of its population. Serosurveys therefore remain an important means of understanding pandemic progression in impoverished regions [53].

## Supporting information

S1 Checklist Inclusivity in global research. (DOCX)

## References

[1] Duong BV, Larpruenrudee P, Fang T, Hossain SI, Saha SC, Gu Y, et al. Is the SARS CoV-2 Omicron Variant Deadlier and More Transmissible Than Delta Variant?. Int J Environ Res Public Health. 2022;19(8):4586. doi: 10.3390/ijerph19084586 35457468 PMC9032753

[2] Binagwaho A, Mathewos K. What explains Africa’s successful response to the COVID-19 pandemic?. Medical News Today. 2020. https://www.medicalnewstoday.com/articles/what-explains-africas-successful-response-to-the-covid-19-pandemic

[3] Frimpong LK, Okyere SA, Diko SK, Abunyewah M, Erdiaw-Kwasie MO, Commodore TS, et al. Actor-network analysis of community-based organisations in health pandemics: evidence from the COVID-19 response in Freetown, Sierra Leone. Disasters. 2022;46(4):903–27. doi: 10.1111/disa.12508 34477244 PMC8652973

[4] casesWorld Health Organization. Africa witnesses longest-running decline in COVID-19 cases. 2023. https://www.afro.who.int/news/africa-witnesses-longest-running-decline-covid-19-

[5] Africa CDC. Africa Joint Continental Strategy for COVID-19 Outbreak. 2020. https://africacdc.org/download/africa-joint-continental-strategy-for-covid-19-outbreak/

[6] Hajissa K, Islam MA, Hassan SA, Zaidah AR, Ismail N, Mohamed Z. Seroprevalence of SARS-CoV-2 Antibodies in Africa: A Systematic Review and Meta-Analysis. Int J Environ Res Public Health. 2022;19(12):7257. doi: 10.3390/ijerph19127257 35742506 PMC9223681

[7] Chisale MRO, Ramazanu S, Mwale SE, Kumwenda P, Chipeta M, Kaminga AC, et al. Seroprevalence of anti-SARS-CoV-2 antibodies in Africa: A systematic review and meta-analysis. Rev Med Virol. 2022;32(2):e2271. doi: 10.1002/rmv.2271 34228851 PMC8420234

[8] Lewis HC, Ware H, Whelan M, Subissi L, Li Z, Ma X, et al. SARS-CoV-2 infection in Africa: a systematic review and meta-analysis of standardised seroprevalence studies, from January 2020 to December 2021. BMJ Glob Health. 2022;7(8):e008793. doi: 10.1136/bmjgh-2022-008793 35998978 PMC9402450

[9] Barrie MB, Lakoh S, Kelly JD, Kanu JS, Squire JS, Koroma Z, et al. SARS-CoV-2 antibody prevalence in Sierra Leone, March 2021: a cross-sectional, nationally representative, age-stratified serosurvey. BMJ Glob Health. 2021;6(11):e007271. doi: 10.1136/bmjgh-2021-007271 34764148 PMC8587532

[10] Murhekar MV, Bhatnagar T, Selvaraju S, Saravanakumar V, Thangaraj JWV, Shah N, et al. SARS-CoV-2 antibody seroprevalence in India, August-September, 2020: findings from the second nationwide household serosurvey. Lancet Glob Health. 2021;9(3):e257–66. doi: 10.1016/S2214-109X(20)30544-1 33515512 PMC7906675

[11] Jalali N, Brustad HK, Frigessi A, MacDonald EA, Meijerink H, Feruglio SL, et al. Increased household transmission and immune escape of the SARS-CoV-2 Omicron compared to Delta variants. Nat Commun. 2022;13(1):5706. doi: 10.1038/s41467-022-33233-9 36175424 PMC9520116

[12] Viana R, Moyo S, Amoako DG, Tegally H, Scheepers C, Althaus CL, et al. Rapid epidemic expansion of the SARS-CoV-2 Omicron variant in southern Africa. Nature. 2022;603(7902):679–86. doi: 10.1038/s41586-022-04411-y 35042229 PMC8942855

[13] Murhekar MV, Clapham H. COVID-19 serosurveys for public health decision making. Lancet Glob Health. 2021;9(5):e559–60. doi: 10.1016/S2214-109X(21)00057-7 33705691 PMC8049585

[14] Koopmans M, Haagmans B. Assessing the extent of SARS-CoV-2 circulation through serological studies. Nat Med. 2020;26(8):1171–2. doi: 10.1038/s41591-020-1018-x 32719488

[15] Yadav AK, Ghosh S, Faujdar DS, Rajmohan KS, Bhalla S, Shekhawat VS, et al. Findings of second multicentric follow-up serosurvey among Health Care Workers in government hospitals. Med J Armed Forces India. 2022. doi: 10.1016/j.mjafi.2022.05.013 PMC9322976 35910399

[16] Statistics Sierra Leone. Population and housing census. 2015.

[17] Africa CDC. Generic protocol for a population-based, age and gender stratified sero-survey study for SARS-CoV-2. 2023.

[18] Richardson ET, Kelly JD, Barrie MB, Mesman AW, Karku S, Quiwa K, et al. Minimally Symptomatic Infection in an Ebola “Hotspot”: A Cross-Sectional Serosurvey. PLoS Negl Trop Dis. 2016;10(11):e0005087. doi: 10.1371/journal.pntd.0005087 27846221 PMC5112953

[19] Kelly JD, Barrie MB, Mesman AW, Karku S, Quiwa K, Drasher M, et al. Anatomy of a Hotspot: Chain and Seroepidemiology of Ebola Virus Transmission, Sukudu, Sierra Leone, 2015-16. J Infect Dis. 2018;217(8):1214–21. doi: 10.1093/infdis/jiy004 29325149 PMC6018898

[20] Kamanga A, Renn S, Pollard D, Bridges DJ, Chirwa B, Pinchoff J, et al. Open-source satellite enumeration to map households: planning and targeting indoor residual spraying for malaria. Malar J. 2015;14:345. doi: 10.1186/s12936-015-0831-z 26376980 PMC4574022

[21] FDA. Serology Test Evaluation Report for “COVID-19 IgG/IgM Rapid Test Cassette” from Hangzhou Biotest Biotech, Co., Ltd. 2020. Available: https://accessdata.fda.gov/cdrh_docs/presentations/maf/maf3252-a001.pdf

[22] World Health Organization. Sierra Leone: the last mile of COVID-19 vaccine delivery. 2023.

[23] Bergeri I, Whelan MG, Ware H, Subissi L, Nardone A, Lewis HC, et al. Global SARS-CoV-2 seroprevalence from January 2020 to April 2022: A systematic review and meta-analysis of standardized population-based studies. PLoS Med. 2022;19(11):e1004107. doi: 10.1371/journal.pmed.1004107 36355774 PMC9648705

[24] Naeimi R, Sepidarkish M, Mollalo A, Parsa H, Mahjour S, Safarpour F, et al. SARS-CoV-2 seroprevalence in children worldwide: A systematic review and meta-analysis. EClinicalMedicine. 2023;56:101786. doi: 10.1016/j.eclinm.2022.101786 36590788 PMC9795163

[25] Wells CR, Stearns JK, Lutumba P, Galvani AP. COVID-19 on the African continent. Lancet Infect Dis. 2020;20(12):1368–70. doi: 10.1016/S1473-3099(20)30374-1 32618281 PMC7202849

[26] Swadling L, Diniz MO, Schmidt NM, Amin OE, Chandran A, Shaw E, et al. Pre-existing polymerase-specific T cells expand in abortive seronegative SARS-CoV-2. Nature. 2022;601(7891):110–7. doi: 10.1038/s41586-021-04186-8 34758478 PMC8732273

[27] Johns Hopkins University School of Medicine Coronavirus Resource Center. Sierra Leone Covid-19 data. Johns Hopkins University Coronavirus Resource Center. 2023. https://coronavirus.jhu.edu/region/sierra-leone

[28] Nwosu K, Fokam J, Wanda F, Mama L, Orel E, Ray N, et al. SARS-CoV-2 antibody seroprevalence and associated risk factors in an urban district in Cameroon. Nat Commun. 2021;12(1):5851. doi: 10.1038/s41467-021-25946-0 34615863 PMC8494753

[29] Moyo S, Simbayi LC, Zuma K, Zungu N, Marinda E, Jooste S, et al. Seroprevalence survey of anti-SARS-CoV-2 antibody and associated factors in South Africa: Findings of the 2020-2021 population-based household survey. PLOS Glob Public Health. 2023;3(9):e0002358. doi: 10.1371/journal.pgph.0002358 37747851 PMC10519586

[30] Konu YR, Condé S, Gbeasor-Komlanvi F, Sadio AJ, Tchankoni MK, Anani J, et al. SARS-CoV-2 antibody seroprevalence in Togo: a national cross-sectional household survey, May-June, 2021. BMC Public Health. 2022;22(1):2294. doi: 10.1186/s12889-022-14794-2 36476149 PMC9730644

[31] Deutou Wondeu AL, Talom BM, Linardos G, Ngoumo BT, Bello A, Ndassi Soufo AM, et al. The COVID-19 wave was already here: High seroprevalence of SARS-CoV-2 antibodies among staff and students in a Cameroon University. J Public Health Afr. 2023;14(1):2242. doi: 10.4081/jphia.2023.2242 36798849 PMC9926561

[32] Kolawole OM, Tomori O, Agbonlahor D, Ekanem E, Bakare R, Abdulsalam N, et al. SARS CoV-2 Seroprevalence in Selected States of High and Low Disease Burden in Nigeria. JAMA Netw Open. 2022;5(10):e2236053. doi: 10.1001/jamanetworkopen.2022.36053 36219441 PMC9554701

[33] Briggs J, Takahashi S, Nayebare P, Cuu G, Rek J, Zedi M, et al. Seroprevalence of Antibodies to SARS-CoV-2 in Rural Households in Eastern Uganda, 2020-2022. JAMA Netw Open. 2023;6(2):e2255978. doi: 10.1001/jamanetworkopen.2022.55978 36790811 PMC9932849

[34] Ndziessi G, Niama RF, Aloumba AG, Peya JM, Ngatse JA, Ngoyomi RA, et al. Seroprevalence of SARS-CoV-2 antibodies in Republic of Congo, February 2022. Epidemiol Infect. 2023;151:e162. doi: 10.1017/S0950268823001425 37800463 PMC10600732

[35] Osman A, Aimone A, Ansumana R, Bogoch I, Gelband H, Colwill K, et al. High SARS-CoV-2 seroincidence but low excess COVID mortality in Sierra Leone in 2020-2022. PLOS Glob Public Health. 2024;4(9):e0003411. doi: 10.1371/journal.pgph.0003411 39255307 PMC11386415

[36] Richardson ET, Barrie MB, Kelly JD, Dibba Y, Koedoyoma S, Farmer PE. Biosocial Approaches to the 2013-2016 Ebola Pandemic. Health Hum Rights. 2016;18(1):115–28. 27781004 PMC5070685

[37] Richardson E. Epidemic illusions: On the coloniality of global public health. 2020.

[38] Frankfurter R, Kardas-Nelson M, Benton A, Barrie B, Dibba Y, Farmer P, et al. Indirect rule redux: the political economy of diamond mining and its relation to the Ebola outbreak in Kono District, Sierra Leone. Rev Afr Polit Econ. 2019;45(158):522–40. doi: 10.1080/03056244.2018.1547188 31772418 PMC6879188

[39] Richardson E. The Instruments of Public Health. In: Wilson Quarterly. 2021 [cited 6 Feb 2024]. Available: https://www.wilsonquarterly.com/quarterly/public-health-in-a-time-of-pandemic/the-instruments-of-public-health

[40] Richardson ET, McGinnis T, Frankfurter R. Ebola and the narrative of mistrust. BMJ Glob Health. 2019;4(6):e001932. doi: 10.1136/bmjgh-2019-001932 31908869 PMC6936462

[41] Nunn N, Wantchekon L. The Slave Trade and the Origins of Mistrust in Africa. American Economic Review. 2011;101(7):3221–52. doi: 10.1257/aer.101.7.3221

[42] Richardson ET, Morrow CD, Ho T, Fürst N, Cohelia R, Tram KH, et al. Forced removals embodied as tuberculosis. Soc Sci Med. 2016;161:13–8. doi: 10.1016/j.socscimed.2016.05.015 27239703

[43] Rodney W. How Europe underdeveloped Africa. London: Verso; 2018.

[44] Mwananyanda L, Gill CJ, MacLeod W, Kwenda G, Pieciak R, Mupila Z, et al. Covid-19 deaths in Africa: prospective systematic postmortem surveillance study. BMJ. 2021;372:n334. doi: 10.1136/bmj.n334 33597166 PMC7887952

[45] Government of Sierra Leone Ministry of Health and Sanitation. COVID-19 vaccination daily situation report. 2023. Available: mohs.gov.sl

[46] Han B, Song Y, Li C, Yang W, Ma Q, Jiang Z, et al. Safety, tolerability, and immunogenicity of an inactivated SARS-CoV-2 vaccine (CoronaVac) in healthy children and adolescents: a double-blind, randomised, controlled, phase 1/2 clinical trial. Lancet Infect Dis. 2021;21(12):1645–53. doi: 10.1016/S1473-3099(21)00319-4 34197764 PMC8238449

[47] Jeewandara C, Kamaladasa A, Pushpakumara PD, Jayathilaka D, Aberathna IS, Danasekara DRSR, et al. Immune responses to a single dose of the AZD1222/Covishield vaccine in health care workers. Nat Commun. 2021;12(1):4617. doi: 10.1038/s41467-021-24579-7 34326317 PMC8322137

[48] Jeewandara C, Aberathna IS, Pushpakumara PD, Kamaladasa A, Guruge D, Wijesinghe A, et al. Immune responses to Sinopharm/BBIBP-CorV in individuals in Sri Lanka. Immunology. 2022;167(2):275–85. doi: 10.1111/imm.13536 35758860 PMC11495257

[49] Eyre DW, Lumley SF, Wei J, Cox S, James T, Justice A, et al. Quantitative SARS-CoV-2 anti-spike responses to Pfizer-BioNTech and Oxford-AstraZeneca vaccines by previous infection status. Clin Microbiol Infect. 2021;27(10):1516.e7-1516.e14. doi: 10.1016/j.cmi.2021.05.041 34111577 PMC8180449

[50] Sadoff J, Le Gars M, Shukarev G, Heerwegh D, Truyers C, de Groot AM, et al. Interim Results of a Phase 1-2a Trial of Ad26.COV2.S Covid-19 Vaccine. N Engl J Med. 2021;384(19):1824–35. doi: 10.1056/NEJMoa2034201 33440088 PMC7821985

[51] World Health Organization W. COVID-19 vaccine delivery partnership. 2023.

[52] Harman S, Erfani P, Goronga T, Hickel J, Morse M, Richardson ET. Global vaccine equity demands reparative justice - not charity. BMJ Glob Health. 2021;6(6):e006504. doi: 10.1136/bmjgh-2021-006504 34919057 PMC8215249

[53] Parkins MD, Lee BE, Acosta N, Bautista M, Hubert CRJ, Hrudey SE, et al. Wastewater-based surveillance as a tool for public health action: SARS-CoV-2 and beyond. Clin Microbiol Rev. 2024;37(1):e0010322. doi: 10.1128/cmr.00103-22 38095438 PMC10938902

